# Recent Progress in Mechanism-Based Therapies for *GJB2*-Related Hearing Loss

**DOI:** 10.3390/ijms27104313

**Published:** 2026-05-12

**Authors:** Chengzhi Liu, Xiaohui Wang, Yu Sun

**Affiliations:** 1 Department of Otorhinolaryngology, Union Hospital, Tongji Medical College, Huazhong University of Science and Technology, Wuhan 430074, China; zeroemia@hust.edu.cn (C.L.); wxh0616@hust.edu.cn (X.W.); 2 Shanghai Key Laboratory of Gene Editing and Cell Therapy for Rare Diseases, Fudan University, Shanghai 200031, China; 3 Institute of Otorhinolaryngology, Union Hospital, Tongji Medical College, Huazhong University of Science and Technology, Wuhan 430074, China; 4 Hubei Province Clinic Research Center for Deafness and Vertigo, Wuhan 430022, China

**Keywords:** *GJB2*, hearing loss, mechanism, therapy

## Abstract

*GJB2*-associated hearing loss is the most common form of non-syndromic hereditary deafness worldwide. However, it exhibits significant heterogeneity in terms of both clinical presentation and biological basis. This review focuses on mechanism-oriented therapeutic strategies for *GJB2*-associated hearing loss, investigating how different types of *GJB2* variants correspond to distinct clinical phenotypes and underlying pathogenic mechanisms, and aims to determine appropriate treatments. Current evidence suggests that *GJB2*-associated hearing loss is not solely caused by channel dysfunction resulting from gap junction defects, but rather the result of multiple pathological processes, including impaired *GJB2* transcriptional regulation, cochlear developmental abnormalities, sensory epithelial degeneration and secondary damage pathways such as inflammation. Consequently, emerging therapeutic approaches can be viewed as interventions targeting specific mechanisms, including gene therapy, restoration of protein transport and pharmacological modulation of damage to the cochlear microenvironment. Overall, this review highlights the importance of aligning therapeutic strategies with specific *GJB2* variants, underlying pathogenic mechanisms, and the developmental window during which cochlear injury remains biologically reversible.

## 1. Introduction

Hearing is the foundation of human communication, social activities and cognitive health [1]. Over the past decade, global health data indicate that hearing loss has emerged as one of the leading causes of disability worldwide [2]. According to the most recent analytical estimates from the Global Burden of Disease (GBD) 2021 study, approximately 1.546 billion individuals experience varying degrees of hearing loss, with projections indicating this figure will rise to around 2.45 billion by 2050 [3,4,5].

The etiological spectrum of hearing loss encompasses a wide range of factors, including both hereditary causes and non-genetic exposures such as infections, noise, and ototoxic agents [6,7,8]. In cases of congenital and early-onset hearing impairment, however, genetic factors play an especially pivotal role [9]. More than half of congenital hearing loss cases can be attributed to genetic origins [10,11]. Non-syndromic sensorineural hearing loss (NSHL) exhibits substantial genetic heterogeneity, with more than 120 hearing loss-associated genes, including both syndromic and nonsyndromic genes, having been identified to date [12,13,14]. Among deafness-associated genes, *GJB2* is one of the most common and clinically important genetic causes of nonsyndromic hearing loss, particularly in autosomal-recessive and congenital/early-onset cases [15]. Biallelic pathogenic variants in *GJB2* are responsible for DFNB1-associated autosomal-recessive nonsyndromic hearing loss, whereas selected heterozygous dominant variants are associated with DFNA3A and several syndromic phenotypes involving both hearing loss and skin disease. Mutations in *GJB2* account for up to approximately 50% of inherited recessive non-syndromic hearing loss (NSHL) across multiple populations [16,17]. More recent cohort studies based on next-generation sequencing have similarly confirmed the prominent contribution of *GJB2* to early-life hearing loss. In a large Canadian pediatric cohort, *GJB2* was the most prevalent causative gene, accounting for 28 of 73 genetically confirmed cases (28/73; 38.4%), while a 2024 study of 106 sporadic hearing loss cases from the United Arab Emirates identified *GJB2* variants in 24 affected individuals (24/106; 22.6%) [18,19]. In this review, we draw upon recent advances in *GJB2*-related hearing loss research to explore mechanistic-level therapeutic strategies, thereby providing references for expanding current treatment strategies and informing feasible future therapeutic pathways of *GJB2*-associated hearing impairment.

## 2. Structure and Function of the *GJB2* Gene

*GJB2* encodes connexin 26 (Cx26), which is highly expressed in multiple non-sensory cochlear cell populations, including supporting cells, sulcus and limbal cells, spiral ligament fibrocytes, and basal cells of the stria vascularis, together with other epithelial and connective tissue cells that collectively form an extensive cochlear gap junction network [20,21]. Within this network, Cx26 assembles into intercellular channels that play critical roles in ionic homeostasis [22,23], metabolic coupling, and local signal transduction [24,25]. Pathogenic variants of *GJB2* represent naturally occurring experimental models, linking molecular defects to clinical phenotypes through their impact on channel assembly, permeability, trafficking, or regulatory function [26,27,28]. These manifestations range from congenital severe-to-profound hearing loss to late-onset mild hearing impairment [29,30,31]. The spectrum of variants in this gene is heterogeneous, encompassing truncating, missense, and splicing alterations, each corresponding to distinct molecular pathological pathways [32,33]. This suggests that the underlying mechanisms involve diverse modes of dysfunction, thereby providing a rationale for the development of mechanism-based therapies rather than mere symptomatic management.

Cx26 does not function in isolation in the cochlea, but rather within a broader connexin network in which its principal partner is Cx30, the protein encoded by *GJB6* [34]. The two proteins are widely co-expressed in cochlear supporting-cell gap junction systems, colocalize within the same junctional plaques, and can co-assemble into heteromeric connexons as well as functionally distinct intercellular channels [35]. This heteromeric organization is functionally important because mixed Cx26/Cx30 channel composition shapes pore properties in ways that influence biochemical coupling more strongly than electrical conductance alone [36,37,38]. In this setting, the local Cx26:Cx30 ratio can help preserve efficient ionic coupling while tuning the permeability of small metabolites and second messengers across cochlear regions [39,40]. In mixed Cx26/Cx30 channels, a higher Cx26 fraction generally favors permeability to anionic tracers and other small anionic solutes, whereas a higher Cx30 fraction tends to reduce such permeability by altering the electrostatic microenvironment and pore constriction profile [41,42,43]. As a result, intercellular signaling pathways, including purinergic and Ca^2+^-related signaling, may exhibit spatially restricted and temporally dynamic propagation during development and under stress conditions [44]. Therefore, the functional consequences of Cx26 deficiency should not be interpreted solely as a loss of a single connexin, but rather in the context of altered Cx26–Cx30 channel composition and coupling properties. This framework helps explain phenotypic variability in DFNB1-related hearing loss and is also relevant to the interpretation of Cx26-targeted rescue strategies [45].

## 3. Genetic Epidemiology and Clinical Spectrum of *GJB2*-Related Hearing Loss

### 3.1. Genetic Epidemiology

Pathogenic *GJB2* variants are among the most common causes of autosomal-recessive non-syndromic hearing loss (AR-NSHL), yet their allele spectra are strongly ancestry-specific. Over one hundred pathogenic or likely pathogenic coding variants have been reported, with marked population specificity [46]. Accordingly, matching panels to local allele spectra improves diagnostic yield, especially in regions dominated by a limited number of recurrent DFNB1 alleles. In East and Southeast Asia, founder and recurrent variants such as c.235delC and c.109G>A (p.V37I) recur at a high frequency, whereas c.35delG predominates in many European populations. Large-scale newborn and carrier screening programs in China and Japan further indicate that a small number of recurrent *GJB2* variants explain a substantial proportion of hereditary deafness, making DFNB1 a practical target for precision screening and prevention. Key population-specific alleles and their screening relevance are summarized in Table 1.

Beyond recurrent coding alleles, the mutational spectrum of the DFNB1 locus also includes non-coding and structural variants that can contribute to unresolved or apparently monoallelic *GJB2* cases. Recurrent deletions spanning the *GJB6*–CRYL1 interval, including del(*GJB6*-D13S1830) and del(*GJB6*-D13S1854), together with additional upstream copy number variants identified through genome sequencing, can reduce *GJB2* expression, often together with *GJB6*, through disruption of cis-regulatory elements rather than simple *GJB6* haploinsufficiency [58,59]. Analyses of overlapping deletion intervals have localized a critical upstream regulatory region largely within CRYL1, providing a mechanistic explanation for cases in which only a single coding *GJB2* variant is initially detected [60]. These findings indicate that the genetic architecture of DFNB1 extends beyond exon-level sequence changes and support the inclusion of copy number and regulatory variant assessment in molecular diagnostic workflows.

### 3.2. Clinical Spectrum

#### 3.2.1. Genotype–Phenotype Correlation and Clinical Variability in Nonsyndromic *GJB2*-Related Hearing Loss

Clinically, most pathogenic *GJB2* alleles are point mutations or small insertions and deletions, yet the associated hearing phenotype is highly variable [61,62]. Across cohorts, a broad genotype–phenotype correlation has nevertheless been observed: biallelic truncating variants, including frameshift, nonsense, and canonical splice-site mutations, are more often associated with prelingual severe-to-profound sensorineural hearing loss, whereas biallelic non-truncating variants more commonly underlie milder phenotypes ranging from mild to moderate impairment [63,64]. In pediatric series, *GJB2*-associated hearing loss most often presents during infancy or early childhood, and earlier onset is generally associated with a higher likelihood of profound impairment [65,66]. This trend is also reflected in several recurrent *GJB2* alleles commonly encountered in clinical practice. Among truncating genotypes, homozygous c.35delG is typically associated with a particularly severe phenotype, and c.235delC, a major recurrent allele in East Asian populations, is likewise frequently linked to congenital or early-onset severe hearing impairment. By contrast, recurrent non-truncating variants such as p.V37I (c.109G>A), p.M34T, and p.L90P are more often associated with mild-to-moderate hearing loss. Representative genotype classes, corresponding nucleotide changes, and their typical phenotypic associations are summarized in Table 2.

This correlation is robust but not absolute. Even among genotypes classified as severe, phenotypic expression can vary, suggesting modulation by genetic background and cochlear network context [67,68]. Clinical series have described families in which biallelic truncating genotypes present with postlingual or moderate impairment, underscoring the relevance of modifiers such as *GJB6* regulatory coupling and other connexin-network determinants [69,70]. Conversely, common missense alleles such as p.V37I often exhibit incomplete penetrance and heterogeneous trajectories, with outcomes ranging from normal hearing to bilateral mild-to-moderate loss of variable configuration and progression [71,72]. Collectively, *GJB2* coding variation supports a spectrum model rather than a single stereotyped phenotype, and genotype-informed stratification is most informative when interpreted alongside modifiers and life-course context.

**Table 2:** Representative genotype classes, nucleotide changes, and clinical spectrum in nonsyndromic DFNB1-associated hearing loss.

Genotype Class	Nucleotide Changes	General Phenotypic Trend	Sources of Variability
Truncating coding variants [73,74]	c.35delG, c.235delC, c.167delT, c.71G>A, c.299_300delAT	Often prelingual severe to profound sensorineural hearing loss	Genetic background and cochlear network context can shift severity and age at onset
Canonical splice-site variants [75,76]	c.−23+1G>A, c.−22-2A>C	Frequently severe, with strong population enrichment in some groups	Expressivity may vary across families and cohorts
Non-truncating coding variants [50]	c.109G>A, c.101T>C, c.269T>C	Often mild to moderate or context-dependent phenotypes	Incomplete penetrance and heterogeneous trajectories are common for recurrent missense alleles
Mixed severity biallelic genotypes [67]	c.35delG/c.109G>A; c.35delG/c.101T>C; c.235delC/c.109G>A	Broad spectrum, often intermediate but not predictable	Modifier burden and life course context can move the phenotype in either direction
DFNB1 regulatory or structural variants [77,78]	del(*GJB6*-D13S1830); del(*GJB6*-D13S1854); upstream DFNB1 cis-regulatory deletions	Can mimic monoallelic *GJB2* results on exon sequencing	Cis regulatory disruption may reduce *GJB2* expression and explain unresolved cases

#### 3.2.2. Syndromic Phenotypes Associated with Specific *GJB2* Variants

Although most *GJB2*-associated hearing loss is nonsyndromic, a smaller subset presents as syndromic disease accompanied by cutaneous abnormalities. These disorders are typically caused by heterozygous dominant missense variants rather than the biallelic truncating alleles that underlie classic DFNB1-associated deafness. Reported syndromic phenotypes include palmoplantar keratoderma with deafness, Vohwinkel syndrome, Bart–Pumphrey syndrome, and keratitis–ichthyosis–deafness (KID) syndrome, all of which illustrate the pleiotropic consequences of specific Cx26 substitutions. Functionally, these variants often act through mechanisms beyond simple loss of channel function, including abnormal hemichannel activity, disrupted gap junction communication, and dominant-negative or trans-dominant interference with wild-type Cx26 or co-expressed connexins. Representative syndromic phenotypes, associated variants, and their translational implications are summarized in Table 3.

**Table 3:** Representative syndromic phenotypes associated with dominant or pleiotropic *GJB2* variants and their translational relevance.

Syndrome	Cardinal Symptom(s)	Hearing Phenotype	Representative *GJB2* Variants	Inheritance Mechanism	Translational Relevance
Palmoplantar keratoderma with deafness (PPK + deafness) [79,80,81]	Diffuse palmoplantar keratoderma, sometimes knuckle involvement	Usually congenital or early-onset SNHL; severity variable	R75W, R75Q, H73R, G59A, S183F	Usually AD; often dominant-negative/trans-dominant effects on Cx26/Cx30	A useful bridge phenotype linking skin disease and cochlear dysfunction; relevant to dominant-variant editing strategies
Vohwinkel syndrome [82,83]	Honeycomb PPK, starfish-like keratoses, pseudoainhum/constriction bands	Usually progressive SNHL, often mild to moderate but variable	Y65H, D66H; some reports also include G130V	Usually AD; impaired gap junction function with variant-specific dominant effects	Highlights the overlap between keratoderma and auditory phenotypes; useful for discussing pleiotropy rather than immediate therapy
Bart–Pumphrey syndrome [84,85]	PPK, knuckle pads, leukonychia	Sensorineural HL with variable severity	N54K	AD; defective trafficking and dominant/trans-dominant effects have been reported	Mechanistically informative because it sits between milder keratoderma phenotypes and more severe KID-spectrum disease
Keratitis–ichthyosis–deafness (KID) syndrome [86,87]	Keratitis, ichthyosis/erythrokeratoderma, severe skin barrier disease, infection/cancer risk in some patients	Usually severe congenital SNHL; some variants associated with lethal early disease	D50N, G45E, A88V, G12R, N14K, N14Y, I30N	Usually AD/de novo; gain-of-function hyperactive hemichannels, plus additional dominant effects in some variants	Best current example for mechanism-based therapy: hemichannel blockade (Figure 1), anti-hemichannel mAb delivery, and dominant-variant editing all have preclinical support
Hystrix-like ichthyosis with deafness (HID)/overlap phenotypes [88]	Severe ichthyotic or spiky hyperkeratotic skin changes	Congenital or early-onset SNHL	Often overlaps with D50N-related spectrum	Overlap disorder within the Cx26 syndromic continuum rather than a wholly separate mechanism	Better treated as a spectrum/extreme phenotype than a standalone major entity in a hearing-focused review

At present, the management of syndromic *GJB2*-associated deafness remains largely supportive, with treatment focused on hearing rehabilitation and dermatologic care rather than mutation-directed intervention. However, accumulating mechanistic studies suggest that these disorders may be particularly informative for future precision therapy. In KID syndrome, where hyperactive hemichannels appear to be a major pathogenic driver, topical pharmacologic inhibition has ameliorated epidermal pathology in mouse models, and AAV-mediated delivery of an anti-hemichannel monoclonal antibody has shown substantial therapeutic benefit in vivo. More recently, AAV-mediated adenine base editing restored cochlear gap junction architecture in a dominant *GJB2* R75W syndromic hearing loss model, supporting the broader concept that dominant missense *GJB2* disorders may become tractable targets for allele-specific editing or other mutation-directed strategies. Accordingly, although direct clinical translation remains preliminary, syndromic *GJB2* phenotypes provide an important framework for linking variant-specific mechanism to future therapeutic design.

**Figure 1:**
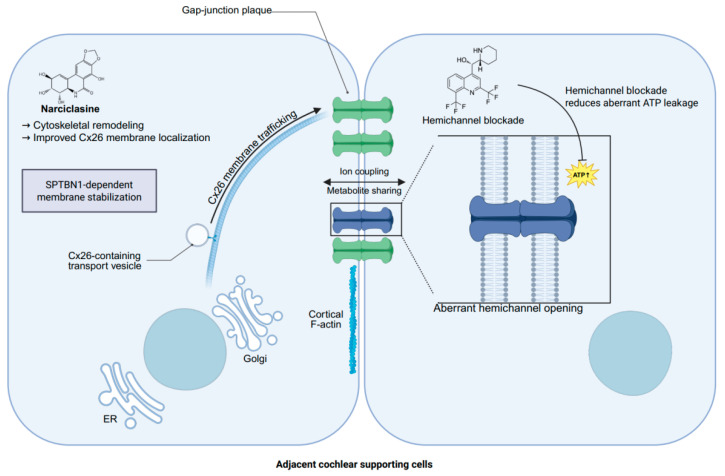
Mechanism-based therapeutic rescue strategies targeting impaired Cx26 trafficking/localization and aberrant hemichannel activity in adjacent cochlear supporting cells.

## 4. Experimental Systems for Studying *GJB2*-Related Hearing Loss

Experimental systems provide the evidentiary basis for connecting *GJB2* variants to cochlear pathology and mechanism-based therapeutic design. Because *GJB2*-related hearing loss involves mutation-specific molecular defects, developmental disruption, supporting-cell network failure, sensory epithelial degeneration, and secondary injury amplification, no single model can fully reproduce the entire disease process. Instead, current understanding relies on complementary systems, including heterologous and cochlear-relevant cell models, ex vivo cochlear organotypic cultures, genetically engineered murine *GJB2* models, and emerging inner-ear organoid platforms. Each system captures a different biological level of the disease and should therefore be interpreted according to its specific strengths and limitations (Table 4).

Cell-based systems are particularly useful for defining variant-level mechanisms. Heterologous expression systems, including HEK293 or HeLa cells and Xenopus oocytes, allow direct assessment of Cx26 protein production, intracellular trafficking, membrane localization, gap junction plaque formation, channel permeability, gating behavior, and aberrant hemichannel activity [28,31,89]. These systems are especially informative for distinguishing loss-of-function variants from dominant-negative or gain-of-function variants, and for identifying mutations that may be amenable to trafficking rescue, channel modulation, or mutant allele suppression [90,91]. However, their interpretive scope is limited because they lack the highly specialized cochlear supporting-cell environment, Cx26/Cx30 network architecture, developmental timing, and tissue-level mechanical context of the inner ear.

More cochlear-relevant cell systems partly address this limitation. Stem-cell-derived Cx26 gap junction-plaque-forming cells and patient-derived iPSC models provide a more humanized platform for studying Cx26/Cx30 plaque assembly, intercellular coupling, and mutation-specific cellular phenotypes [92,93,94]. These models are valuable for mechanistic screening and early drug evaluation, particularly for variants that affect plaque organization, membrane stability, or intercellular communication. Nevertheless, they remain simplified systems and cannot fully reproduce the three-dimensional organ of Corti, lateral-wall physiology, endolymphatic ionic gradients, or long-term auditory function [95].

Ex vivo cochlear organotypic cultures occupy an intermediate position between isolated cell models and in vivo animal systems [96]. They preserve part of the immature cochlear architecture, including local relationships among supporting cells, sensory epithelium, and the developing gap junction network [97,98]. This makes them useful for studying early tissue-level rescue, especially when the therapeutic question is whether *GJB2* delivery can restore Cx26 expression and intercellular coupling before irreversible degeneration has occurred. For example, viral *GJB2* transfer in cochlear organotypic cultures from Cx26-deficient mice restored Cx26 expression and improved gap junction coupling, providing early proof of concept for gene supplementation. However, organotypic cultures cannot fully model mature cochlear mechanics, endocochlear potential, systemic immune responses, vector biodistribution, or long-term ABR and DPOAE outcomes [99].

Murine *GJB2* models remain indispensable for linking molecular pathology to cochlear development, auditory physiology, and therapeutic efficacy. Conditional knockout, knockdown, knock-in, dominant-negative, and inducible models have collectively shown that Cx26 deficiency can impair postnatal organ of Corti maturation, disrupt supporting-cell differentiation, alter active cochlear amplification, promote sensory epithelial degeneration, and trigger secondary inflammatory or stress responses [25,100,101,102]. These models are also essential for evaluating therapeutic timing, vector delivery, cell specificity, dosing, toxicity, and functional outcomes [103]. Importantly, different murine models answer different questions. Early conditional deletion models are powerful for identifying developmental requirements, whereas inducible or partial-loss models may better represent residual cochlear structure and later therapeutic windows [104]. Knock-in models carrying patient-relevant variants are particularly valuable for studying genotype-specific phenotypes, including mild, progressive, dominant-negative, or gain-of-function mechanisms [105]. Therefore, discrepant therapeutic outcomes across murine studies should not be interpreted simply as contradictory results, but as evidence that rescue depends on developmental stage, residual cochlear architecture, target-cell competence, expression level, vector tropism, and the degree of pre-existing sensory epithelial or neural injury [106].

Inner-ear organoids provide a promising but still developing platform for humanized modeling of hereditary hearing loss [107,108]. In principle, organoids may allow patient-specific disease modeling, scalable drug screening, and preliminary evaluation of gene- or RNA-based therapeutic strategies [109,110]. However, current organoid systems do not yet fully reproduce the mature cochlear supporting-cell network, lateral wall and strial physiology, vascular and immune microenvironment, endolymphatic homeostasis, tonotopic organization, or functional auditory output [111,112,113]. For *GJB2*-related hearing loss, this limitation is particularly important because the disease depends heavily on supporting-cell gap junction networks and cochlear microenvironmental regulation rather than on hair cell autonomy alone. Thus, organoids should currently be viewed as complementary platforms for human genetic modeling and preclinical screening, rather than substitutes for murine *GJB2* models or in vivo functional validation.

## 5. Pathogenic Mechanisms and Therapeutic Opportunities in *GJB2*-Related Hearing Loss

### 5.1. Therapeutically Relevant Upstream Defects in Cx26 Expression, Trafficking, and Localization

Upstream defects in Cx26 expression, trafficking, and localization represent one of the most therapeutically actionable entry points in *GJB2*-related hearing loss. At this level, pathogenic variants do not all disrupt cochlear function in the same manner: some primarily reduce Cx26 abundance, whereas others permit protein synthesis but impair its intracellular trafficking, junctional assembly, membrane stability, or channel competence. This mechanistic heterogeneity is important because it means that early pathogenic events may be more amenable to correction than downstream structural degeneration, but also that rescue strategies cannot be applied in a mutation-agnostic fashion. Instead, effective intervention must be matched to the biological level of failure, with expression-restoring approaches being more relevant to protein-deficient alleles and localization- or assembly directed strategies being more relevant to structurally permissive variants. The following sections therefore begin by outlining the major forms of upstream Cx26 dysfunction and then discuss how these distinct defects shape corresponding therapeutic strategies.

#### 5.1.1. Reduced Cx26 Abundance and Impaired Trafficking, Assembly, and Channel Competence

Pathogenic *GJB2* variants can disrupt cochlear function at several proximal steps in the Cx26 life cycle, ranging from protein production to membrane delivery, junctional assembly, and channel function [62,114]. Separating these defects is important because each class implies a different therapeutic logic.

For many truncating variants, including nonsense, frameshift, and canonical splice-site mutations, the primary defect is quantitative. These variants often lead to absent or markedly reduced Cx26 production, thereby severely limiting the formation of functional gap junction channels [115]. Even when translation is not completely abolished, some mutant proteins are unstable and fail to accumulate to levels sufficient for normal intercellular coupling [116,117]. Certain missense variants may also reduce functional Cx26 availability by impairing protein stability or conformational integrity [89]. This defect class provides the clearest rationale for strategies that restore Cx26 abundance, such as gene supplementation, transcript correction, or variant-specific approaches aimed at increasing the amount of functional protein.

A second major defect involves abnormal intracellular trafficking [118]. Newly synthesized Cx26 must undergo proper folding, oligomerization, and delivery to the plasma membrane before it can participate in gap junction plaque formation [119]. Many disease-causing variants are retained within the endoplasmic reticulum, Golgi apparatus, or other intracellular compartments, preventing efficient membrane insertion [120]. For these variants, therapeutic rescue would require more than increasing total Cx26 expression; it would need to improve protein folding, intracellular transport, or escape from aberrant retention pathways.

Some mutant Cx26 proteins can reach the plasma membrane but fail to form normal plaque-like structures at sites of cell–cell contact [90]. This distinction is therapeutically important because membrane localization alone does not guarantee functional intercellular coupling. Variants in this category may require approaches that stabilize Cx26 at the membrane, reinforce junctional scaffolding, or promote proper plaque organization, rather than simply increasing protein abundance [121]. This provides a mechanistic basis for therapeutic strategies aimed at improving membrane retention and junctional assembly.

Even when mutant Cx26 is expressed and delivered to the membrane, channel competence may remain impaired. Pathogenic variants can disrupt connexon assembly, reduce docking efficiency between adjacent hemichannels, and alter channel permeability, selectivity, or gating behavior [122]. Because Cx26 normally interacts closely with Cx30 in the cochlea, these defects may also perturb heteromeric or heterotypic channel function and amplify network-level dysfunction. In selected dominant or syndromic variants, aberrant hemichannel activity may further introduce a toxic gain-of-function component through inappropriate channel opening and membrane leakiness [123]. These variants require a different therapeutic framework: restoring expression alone may be insufficient, and in some contexts, selective suppression of mutant activity or inhibition of pathological hemichannel opening may be more appropriate.

Taken together, upstream *GJB2* defects can be organized into four partially overlapping categories: insufficient Cx26 production, defective trafficking, impaired junctional assembly, and loss or distortion of channel function. This classification clarifies why therapeutic rescue cannot follow a single strategy for all variants. Instead, rational intervention should be matched to the dominant molecular defect, with abundance-restoring, trafficking-correcting, membrane-stabilizing, channel-rescuing, or gain-of-function-suppressing approaches applied according to the specific pathogenic mechanism.

#### 5.1.2. Therapeutic Rescue of Upstream Cx26 Defects

Therapeutic rescue of upstream Cx26 defects should be guided by the biological level at which a given *GJB2* variant fails, because not all pathogenic alleles are amenable to the same corrective strategy. For variants associated with little or no functional protein production, including many truncating and severe splice-site alleles, the most rational upstream objective is not post-translational rescue alone but restoration of Cx26 expression itself. In this setting, gene supplementation has emerged as the clearest proof-of-concept approach. Experimental studies have shown that viral delivery of *GJB2* can restore Cx26 expression and re-establish coupling within the supporting-cell gap junction network, thereby demonstrating that at least part of the upstream molecular deficit is replacement-amenable [97,99]. However, these studies have also clarified an equally important point: histologic or molecular correction does not automatically translate into functional hearing recovery. In vivo, broad restoration of connexin 26 expression may reduce sensory epithelial injury and partially reconstruct intercellular networks while yielding limited or inconsistent ABR improvement [124]. This discrepancy suggests that the therapeutic endpoint is not simply re-expression of Cx26, but restoration of appropriately localized, physiologically integrated Cx26 within the native cochlear supporting-cell network. Accordingly, the design problem in *GJB2* gene therapy is now less about whether Cx26 can be delivered and more about how to deliver it in the right cells, at the right level, and within the right developmental window. Perinatal delivery has shown a clearer benefit than later-stage intervention in several experimental settings, supporting the view that upstream correction becomes less effective once developmental disorganization and secondary degeneration are already established [125]. At the same time, work in more mature cochleae has highlighted a safety ceiling: robust transduction and a strong connexin 26 signal can coexist with absent threshold rescue, and in some cases with new injury, particularly when expression extends beyond the physiologic supporting-cell domain [126,127,128]. These findings argue strongly that successful gene-based rescue will require tight control over cell specificity, expression level, and vector tolerability rather than simple maximization of transduction efficiency. Consistent with this principle, newer strategies using regulatory elements to constrain *GJB2* expression to appropriate cochlear cell populations appear more promising than earlier approaches driven primarily by strong promoters alone, and they support a broader shift toward integrated optimization of capsid, regulatory design, dose, and local immunologic safety [129,130,131]. Importantly, the outcomes of *GJB2* gene supplementation vary substantially across experimental models. Ex vivo organotypic cultures and neonatal conditional knockout models tend to demonstrate more direct restoration of Cx26 expression and intercellular coupling, whereas inducible or more mature in vivo models often show weaker functional recovery despite detectable connexin expression [97]. These discrepancies likely reflect differences in the developmental stage, residual cochlear architecture, target-cell competence, vector tropism, expression level, route of delivery, and the degree of pre-existing sensory epithelial or neural injury [99]. Therefore, gene therapy outcomes should not be interpreted as uniformly successful or unsuccessful, but rather as model-dependent evidence defining the biological conditions under which Cx26 restoration remains functionally meaningful. Representative studies of gene supplementation for expression-deficient *GJB2* variants are summarized in Table 5.

For variants that retain at least partial structural competence, especially selected missense alleles, a second therapeutic route is to improve the amount of Cx26 that successfully reaches and persists at intercellular junctions. In these cases, the central defect may lie less in complete absence of protein than in impaired trafficking, unstable membrane residence, or defective plaque formation. Current evidence supporting this strategy remains limited but is no longer purely conceptual. Recent work has shown that normal Cx26 membrane localization depends on an intracellular transport network involving microtubules, actin microfilaments, and the Golgi apparatus, and has further identified SPTBN1 as a membrane-proximal factor required for maintaining Cx26 at the plasma membrane, thereby nominating junctional stabilization itself as a therapeutically relevant target. In the same study, the small molecule Narciclasine promoted cytoskeletal development, enhanced membrane localization of several cytoplasm-retained Cx26 mutants, improved coupling-related readouts, and was accompanied by partial rescue of hearing loss and hair cell degeneration in murine *GJB2* models (Figure 1). Earlier cell-based work also showed that trafficking-defective mutants such as G59A and D66H could be partially rescued through co-expression with wild-type Cx26 or Cx30, providing proof of principle that compatible connexin assembly can, in some contexts, restore junctional delivery of retained mutants [96]. Together, these findings suggest that localization-directed rescue is most plausible for a restricted subset of variants that are produced but misprocessed or mislocalized, whereas mutants with severe docking, permeability, gating, or toxic hemichannel defects are less likely to benefit from improved membrane targeting alone.

### 5.2. Developmental Disruption and Rescue of Cochlear Maturation

Beyond the upstream molecular defects discussed above, *GJB2*-related hearing loss must also be understood as a disorder of disrupted cochlear maturation. In this context, Cx26 deficiency is important not only because it impairs connexin expression or intercellular coupling, but because it interferes with the developmental programs that shape the organ of Corti and the supporting-cell network during a critical period of postnatal maturation. This developmental perspective has major therapeutic implications. Once abnormal maturation becomes structurally embedded, later molecular correction may be unable to fully restore normal cochlear architecture or function, even when connexin expression is partially recovered. Accordingly, this section focuses on two related questions: first, how Cx26 contributes to normal cochlear maturation, and second, why the timing of intervention is a central determinant of whether developmental rescue remains biologically achievable.

#### 5.2.1. Developmental Roles of Cx26 in Cochlear Maturation

Cx26 plays a fundamental developmental role in cochlear maturation, not merely by maintaining intercellular coupling in the mature ear, but by actively supporting the structural and functional assembly of the immature cochlea [134,135]. During late embryonic and early postnatal development, Cx26 expression emerges in a spatially organized manner within non-sensory epithelial and connective tissue compartments, including the greater epithelial ridge, pillar cells, Deiters’ cells, Claudius cells, and other supporting-cell populations, whereas sensory hair cells themselves do not express Cx26 [136,137]. This distribution suggests that the developmental importance of Cx26 lies primarily in the supporting-cell network that coordinates the local microenvironment required for organ of Corti maturation [138].

Evidence from conditional *GJB2*-deficient models indicates that Cx26 is indispensable for the postnatal remodeling events that prepare the cochlea for hearing onset. When Cx26 is absent, the organ of Corti may appear grossly formed at birth, yet its subsequent maturation is profoundly disturbed [139,140]. Key architectural landmarks, particularly the opening of the tunnel of Corti and the formation of Nuel’s space, fail to develop normally, indicating that Cx26 is required not simply for tissue maintenance but for the execution of the maturation program itself [100,141]. These abnormalities are accompanied by arrested differentiation of supporting cells, subsequent degeneration of Claudius cells and outer hair cells, and later secondary loss of spiral ganglion neurons [142,143]. Importantly, the developmental actions of Cx26 are also spatially specific. High-resolution analyses suggest that pillar cells and Deiters’ cells represent especially critical nodes, as their junctional organization appears closely linked to the structural integrity of the sensory epithelium and the long-term survival of outer hair cells [144]. Thus, Cx26 should be viewed as a developmental organizer of the cochlear supporting-cell syncytium, helping shape the architecture, intercellular coordination, and microenvironmental stability needed for normal cochlear maturation [145]. This developmental perspective also provides the mechanistic basis for why early restoration of Cx26 is likely to be more effective than delayed intervention.

More recently, an emerging study has even proposed that Cx26 may localize to the nucleus of supporting cells and directly influence transcription of genes involved in cochlear structural development. This study demonstrates that Cx26 acts as a regulatory factor that directly modulates the TSPANC8/ADAM10 axis, controlling the opening of the cochlear Corti’s canal and the maturation of cochlear structures, thereby influencing the development of hearing [146].

#### 5.2.2. Therapeutic Window, Developmental Reversibility, and Early Rescue

In *GJB2*-related hearing loss, the therapeutic significance of developmental timing lies not simply in treating earlier, but in intervening while cochlear maturation remains biologically rescuable. Cx26 deficiency can derail the postnatal developmental program of the organ of Corti, leading to disturbed supporting-cell differentiation, failure of tunnel of Corti and Nuel’s space opening, and broader disorganization of the epithelial architecture required for later cochlear amplification [133]. Once these abnormalities become structurally embedded, subsequent molecular correction may no longer be sufficient to reconstruct the original maturation trajectory [132]. From a therapeutic perspective, the relevant question is therefore not only whether Cx26 expression can be restored, but whether restoration occurs during the window in which developmental programs can still be re-engaged [99].

Available evidence strongly supports the existence of such a critical window. In early postnatal explant systems and neonatal in vivo models, *GJB2* supplementation has been shown to restore intercellular coupling and re-establish supporting-cell gap junction networks, indicating that immature cochlear tissue retains substantial developmental plasticity [97]. In several perinatal or very early postnatal paradigms, intervention has also been associated with improved preservation of sensory epithelial structure, attenuation of secondary neural injury, and in some settings measurable recovery of auditory brainstem responses [106]. These findings suggest that early rescue is effective not merely because it precedes extensive degeneration, but because it coincides with a stage at which the supporting-cell syncytium and cochlear architecture have not yet irreversibly diverged from normal development [147].

By contrast, later intervention appears much more constrained. In more mature cochleae, the connexin signal may be partially restored without meaningful recovery of hearing, implying that histologic or molecular correction alone does not guarantee functional rescue once developmental defects have already propagated into tissue architecture and cochlear mechanics [148]. Timing is not the only determinant of outcome, however. Therapeutic performance is also shaped by vector tropism, delivery route, expression level, and cellular specificity [99]. In some experimental settings, ectopic or excessive connexin expression has introduced additional injury rather than benefit, underscoring that developmental rescue requires not only early delivery but also biologically appropriate targeting [102]. The goal is not indiscriminate re-expression of Cx26, but restoration of the right protein in the right cells at the right developmental stage.

This stage-dependent view provides a useful framework for distinguishing prevention, reversal, and attenuation of *GJB2*-related cochlear pathology. In principle, prenatal intervention would be the closest strategy to developmental prevention. Because the pathogenic *GJB2* defect is already present during fetal life and Cx26 expression begins during embryonic cochlear development, prenatal delivery could theoretically restore connexin-dependent signaling before supporting-cell differentiation, tunnel of Corti formation, and sensory epithelial organization deviate from their normal trajectory [149,150,151,152]. This window may be particularly relevant for severe congenital genotypes in which cochlear maldevelopment begins before birth [153,154]. However, prenatal therapy should not be interpreted as an immediately available solution. It remains constrained by the timing and accuracy of fetal diagnosis, procedural risk to the fetus and pregnancy, incomplete control of vector biodistribution, ethical considerations, and uncertainty regarding the competence of fetal cochlear target cells at different developmental stages [155,156]. Thus, prenatal intervention is best viewed at present as a conceptually attractive but technically and ethically demanding strategy aimed at preventing irreversible maldevelopment rather than repairing established hearing loss.

Early postnatal intervention currently represents the most realistic therapeutic window, because it can be linked to newborn hearing screening, rapid genetic diagnosis, and early cochlear management while the supporting-cell network and sensory epithelium may still retain structural competence [157]. Experimental studies support this view. In organotypic cochlear cultures, viral *GJB2* transfer restored Cx26 expression and improved intercellular coupling, demonstrating that the primary molecular and coupling defects can be corrected in immature tissue contexts [97]. In neonatal conditional *GJB2* models, gene supplementation re-established the gap junction network and reduced epithelial injury, although auditory brainstem response recovery has been limited or inconsistent in some settings [99]. More favorable outcomes after perinatal delivery than after adult-stage intervention further suggest a narrow developmental rescue window in which preservation of cochlear architecture and partial functional recovery remain achievable [132]. Importantly, early rescue need not depend exclusively on direct connexin replacement. Complementary strategies that support broader maturation programs may also be useful while cochlear architecture is still being shaped. For example, T3 can promote tunnel of Corti opening by upregulating ADAM10, thereby reducing hair cell loss and partially improving hearing impairment caused by Cx26 deficiency [157]. Nevertheless, such maturation-supporting approaches should currently be regarded as exploratory adjuncts rather than substitutes for genotype-directed therapy.

By contrast, later postnatal or mature-cochlea intervention appears less capable of true structural reversal. In more mature inducible models, Cx26 expression can be partially restored without meaningful auditory recovery, implying that molecular correction alone may fail once developmental abnormalities, hair cell dysfunction, or neural injury have become structurally fixed [133,148]. This does not mean that later intervention is useless. Rather, its goal may shift from reversing maldevelopment to slowing progression, preserving residual hair cells or spiral ganglion neurons, reducing secondary inflammation, or improving the microenvironment in which remaining cochlear elements operate.

These temporal constraints also shape the expected value of RNA-based approaches. For recessive expression-deficient alleles, RNA-based transcript correction or splice modulation would still need to occur before irreversible developmental or degenerative injury has accumulated [158]. For dominant-negative or gain-of-function variants, allele-specific RNA interference may offer a different therapeutic logic, because suppressing the mutant transcript could reduce toxic interference with wild-type Cx26 or Cx30 [159]. This strategy may be especially relevant for progressive dominant phenotypes, where residual cochlear structure may persist long enough to provide a broader postnatal intervention window [160]. Nevertheless, RNA-based therapies are also unlikely to restore normal hearing once the sensory epithelium, supporting-cell architecture, or spiral ganglion population has already been lost [161]. Therefore, the therapeutic window in *GJB2*-related hearing loss should be defined not only by chronological age, but also by variant mechanism, residual cochlear architecture, target-cell competence, and whether the intended intervention aims to prevent, reverse, or merely attenuate disease progression.

### 5.3. Sensory Epithelial Degeneration and Cell-Protective Interventions

Although the initiating lesion in *GJB2*-related hearing loss resides primarily within the connexin-based supporting-cell network, one of its most clinically and therapeutically consequential downstream outcomes is progressive degeneration of the sensory epithelium. This process extends beyond simple hair cell disappearance and includes region-specific epithelial destabilization, selective outer hair cells vulnerability, and secondary injury that can ultimately constrain functional rescue. Importantly, once sensory epithelial damage becomes established, the opportunity for recovery may depend not only on correcting the upstream connexin defect, but also on limiting the cellular programs that execute tissue loss. The following two subsections therefore address this downstream phase from complementary perspectives: first, the spatial and mechanistic patterns by which sensory epithelial degeneration emerges across *GJB2*-deficient models; and second, the cell-death pathways and protective interventions that may help preserve cochlear structure and prolong the window for functional salvage.

#### 5.3.1. Patterns and Mechanisms of Sensory Epithelial Degeneration

Sensory epithelial degeneration in *GJB2*-related hearing loss is better viewed as a spatially patterned, model-dependent downstream phenotype than as a uniform initiating lesion. Across Cx26-deficient mouse models, the earliest abnormalities often arise within the non-sensory supporting-cell network, and the subsequent epithelial injury varies substantially with genetic design and developmental timing. In the OtogCre null model, cell death initially involved supporting cells adjacent to the inner hair cell region rather than indiscriminate early hair cell collapse. Early postnatal knockdown models likewise showed that malformed Deiters’ cell phalangeal processes reduced pillar-cell microtubules, and failure of normal organ of Corti architecture can precede or accompany later sensory epithelial injury [145]. By contrast, in later or mature inducible loss models, cochlear dysfunction may emerge in the relative absence of rapid structural degeneration, with reduced DPOAEs, altered outer hair cell electromotility, and survival of hair cells for weeks to months after Cx26 reduction [133]. A similar dissociation is evident in the *GJB2* 35delG/35delG model, in which mice are already profoundly deaf and lack DPOAEs at P14 while hair cells and adjacent supporting cells remain largely intact, whereas by P35 they show partial outer hair cell loss with relative preservation of supporting cells [100]. Together, these findings argue that overt hair cell degeneration is common but not invariably the earliest driver of dysfunction [134,162]. When degeneration becomes morphologically evident, it is typically selective rather than diffuse: outer hair cells are often more vulnerable than inner hair cells, and their injury is accompanied by plasma membrane deformation, loss of the characteristic wavy cortical lattice surface, and abnormal caveolin-2 accumulation [163]. Local macrophage recruitment further accompanies these lesions and tracks outer hair cell loss and CX3CL1 upregulation, supporting inflammatory remodeling as a secondary amplifier of sensory epithelial damage rather than a proven initiating event [164].

#### 5.3.2. Cell Death Pathways and Protective Interventions

Rather than converging on a single uniform execution program, sensory epithelial degeneration in *GJB2*-related hearing loss appears to involve multiple partially overlapping cell-death pathways whose relative importance varies across models, cochlear regions, and disease stages. Early work in conditional *GJB2* knockout mice identified typical apoptotic morphology in outer hair cells, with OHC loss becoming evident between P12 and P21 and clear apoptotic surface changes observed around P18 [165]. These findings support the involvement of apoptosis in at least part of the degenerative process, but they do not justify reducing all epithelial injury in Cx26-deficient cochleae to a single canonical apoptotic mechanism [134]. This more cautious interpretation is reinforced by later mechanistic work showing that the miR-27a/sgk1 axis contributes to cochlear sensory epithelial apoptosis in Cx26 knockout mice, and that suppression of miR-27a attenuates apoptosis while restoring sgk1 expression [166]. Together, these studies indicate that apoptosis is a genuine component of *GJB2*-related epithelial injury, yet one that is best viewed as context-dependent rather than universally dominant. In a mouse model of progressive hair cell death induced by conditional *GJB2* knockout, hair cell death occurs via the parthanatos pathway. This is manifested by the accumulation of PARP-1 and the nuclear translocation of the AIF in hair cells [167].

A more therapeutically useful framework is to place oxidative injury and PARP1-dependent cell death downstream of the initiating connexin defect. Although much of the detailed biochemical evidence for PARP1-mediated injury derives from oxidative stress models rather than exclusively from mutant hair cells themselves, this line of work has become directly relevant to *GJB2* pathology through the demonstration that PARP inhibition can rescue hearing and hair cell impairment in Cx26-null mice, supporting the involvement of a parthanatos-like pathway as more than a speculative bystander mechanism [104,168,169,170]. In parallel, a recent study reported that verapamil and nimodipine preserved auditory function and reduced outer hair cell loss in Cx26-cKO mice, nominating calcium overload as another potentially actionable contributor to downstream epithelial death [171]. Taken together, these observations suggest that the most informative therapeutic targets may lie not in nonspecific anti-apoptotic strategies, but in more coherent intervention nodes that interrupt regulated death execution after the primary connexin defect has already destabilized the tissue.

Protective interventions in this setting can therefore be divided into two broad categories. The first includes pathway-directed approaches that more directly interfere with regulated epithelial death, exemplified by PARP inhibition and, more recently, calcium-channel blockade [172]. The second includes adjunct neuro-protective strategies that do not primarily prevent hair cell death itself but may still preserve downstream neural elements [161]. In this regard, BDNF is best interpreted as a supportive example rather than a central epithelial rescue strategy, because its clearest demonstrated benefit in *GJB2* models has been rescue of spiral ganglion neurons, particularly in the basal turn, rather than direct prevention of sensory-hair cell degeneration. By contrast, broader anti-inflammatory or stress-modulating interventions such as dexamethasone are conceptually better discussed separately as modulation of downstream injury amplification rather than as a direct blockade of a specific death-execution program [173].

### 5.4. Cochlear Homeostatic Disruption and Targeted Intervention

In *GJB2*-related hearing loss, disruption of cochlear homeostasis represents an important downstream consequence of connexin deficiency and provides a plausible link between supporting-cell network dysfunction and progressive functional decline [174,175]. Once cochlear gap junction coupling is impaired, the supporting-cell network becomes less capable of coordinating ionic buffering, metabolic exchange, and local intercellular signaling, thereby compromising the conditions required for normal hair cell function [176]. Within this framework, potassium dysregulation, abnormal calcium handling, and impaired active cochlear amplification can be viewed as related consequences of supporting-cell homeostatic failure rather than as wholly independent abnormalities [177]. This interpretation is therapeutically relevant because it broadens the focus beyond upstream molecular correction alone and supports the idea that preserving the local environment of the organ of Corti may help limit secondary epithelial injury when direct restoration of connexin function remains incomplete.

#### 5.4.1. Homeostatic Breakdown and Ionic Dysregulation in the Cochlear Supporting-Cell Network

At the tissue level, ionic dysregulation in *GJB2*-related hearing loss is better understood as a failure of a multicellular buffering system than as the isolated loss of a single channel function. In the normal cochlea, Cx26 and Cx30 are the predominant connexins in non-sensory cells, coupling supporting cells of the organ of Corti with epithelial and connective-tissue networks that extend toward the spiral limbus, spiral ligament, and stria vascularis [175]. Through this syncytial organization, the cochlea maintains not only intercellular electrical communication, but also the coordinated redistribution of ions, metabolites, and small signaling molecules required to preserve the highly specialized extracellular environment around hair cells [178]. Once Cx26 expression is reduced or connexin channel function is compromised, this cooperative homeostatic capacity becomes unstable, and the resulting pathology is more plausibly framed as network-level dysregulation of the cochlear microenvironment [179].

One major manifestation of this breakdown is disturbance of potassium handling. The classical concept that cochlear gap junction networks participate in K^+^ recirculation remains an important conceptual framework, because it captures the anatomical continuity between supporting cells, lateral wall fibrocytes, and the strial machinery that sustains endolymphatic composition [180,181,182]. However, the pathogenic consequences of Cx26 deficiency do not need to be interpreted as uniform interruption of every step in a rigid linear recycling pathway [183]. A more defensible view is that impaired intercellular coupling weakens spatial buffering and ionic redistribution within the supporting-cell and lateral-wall networks, thereby reducing the cochlea’s ability to stabilize local extracellular K^+^ loads and maintain a resilient electrochemical milieu under the stress of ongoing sensory transduction [134]. This interpretation also fits the heterogeneity of *GJB2*-related phenotypes, including conditions in which severe early hair cell loss or overt collapse of the endocochlear potential is not the dominant initial event [184].

Calcium dysregulation likely represents a second, and therapeutically more tractable, arm of this homeostatic failure. In the cochlear supporting-cell network, connexin hemichannels and assembled gap junction channels cooperate to support ATP-dependent intercellular signaling and the spread of Ca^2+^-mobilizing messengers [185,186,187,188]. Accordingly, connexin dysfunction is not limited to reduced ionic conductance in the narrow electrical sense, but may also impair the propagation of biochemical signals that normally constrain local Ca^2+^ dynamics across the sensory epithelium [189]. This point is mechanistically important because some deafness-associated Cx26 variants can preserve gross electrical coupling while selectively disrupting permeability to signaling molecules such as IP_3_, indicating that metabolic and second-messenger uncoupling may be pathogenic even when simple conductance measurements appear less abnormal [190]. In this context, Cx26 deficiency may favor disordered Ca^2+^ handling, abnormal stress signaling, and progressive vulnerability of the sensory epithelium, providing a mechanistic bridge between supporting-cell network failure and downstream cellular injury [191,192].

Taken together, these observations suggest that the core lesion in this subsection is not merely loss of connexin expression, but destabilization of the ionic and signaling microenvironment on which cochlear function depends [193]. This framing is useful for the discussion that follows, because once cochlear pathology is interpreted as microenvironmental failure involving both K^+^ buffering and Ca^2+^ dysregulation, therapeutic strategies no longer need to be limited to upstream gene replacement alone but can also include interventions aimed at preserving the local milieu required for hair cell function and survival.

#### 5.4.2. Active Amplification Failure and Microenvironment-Targeted Rescue

Outer hair cell dysfunction in *GJB2*-related hearing loss should be considered in the context of the surrounding supporting-cell network, because active cochlear amplification relies on the mechanical, ionic, and metabolic environment that these neighboring cells help maintain [194]. Experimental findings support a close functional relationship between the cochlear supporting-cell network and outer hair cell amplification [195,196]. Disruption of Cx26 in supporting-cell populations associated with the outer hair cell region has been linked to altered electromotility, reduced distortion product otoacoustic emissions, and preferential impairment of high-frequency hearing [134]. In postnatal inducible models, these functional abnormalities can emerge even when gross cochlear development is initially preserved and overt early hair cell loss is not yet apparent, suggesting that compromised active amplification may represent an early consequence of supporting-cell network dysfunction [184]. Framed in this way, the key lesion is not simply disappearance of outer hair cells, but deterioration of the local microenvironment that normally allows them to operate as cochlear amplifiers.

These findings warrant a cautious mechanistic interpretation. Although endocochlear potential is reduced in postnatal inducible Cx26-deficient mice, this change does not parallel the progressive pattern of hearing decline [197]. This argues against explaining the phenotype solely by endocochlear potential reduction or by a rigid potassium-recycling defect, and instead supports the view that dysfunction of the supporting-cell network can compromise active cochlear amplification before overt sensory epithelial degeneration becomes evident.

From a therapeutic perspective, direct pharmacologic correction of potassium-handling abnormalities remains poorly developed. By contrast, calcium-directed protection has begun to show more explicit proof of concept. In a recent Cx26-cKO model, intracellular calcium accumulation was observed in cochlear hair cells, and treatment with verapamil or nimodipine ameliorated hearing loss and reduced outer hair cell loss, particularly in the basal turn [171]. These data do not establish calcium dysregulation as the sole downstream mechanism, but they do suggest that limiting ionic stress may help attenuate sensory epithelial injury when the upstream connexin defect is not yet fully corrected.

### 5.5. Secondary Pathogenic Pathways and Therapeutic Modulation

Beyond the more direct consequences of Cx26 deficiency on cochlear development, sensory epithelial integrity, and tissue homeostasis, accumulating evidence suggests that *GJB2*-related hearing loss may also involve broader disturbances in cellular regulation and secondary injury amplification [105]. These processes do not simply represent passive by-products of the initiating connexin defect [198]. Rather, they may influence how cochlear injury evolves over time by reshaping stress responses, redox balance, immune activity, and the molecular programs that govern epithelial vulnerability [101]. This broader framework is therapeutically relevant because it identifies a layer of pathology that is not fully captured by structural rescue or restoration of intercellular coupling alone. Even when the primary defect cannot be completely corrected, modulation of these downstream processes may still help limit collateral damage, stabilize the injured cochlear environment, and improve the conditions under which more definitive restorative interventions can succeed.

#### 5.5.1. Transcriptional Dysregulation and Secondary Injury Amplification

The pathogenic reach of Cx26 deficiency may extend beyond canonical gap junction failure to broader disturbances in cellular state regulation. In addition to impairing intercellular exchange, loss of Cx26 has been shown to disrupt miRNA-mediated genetic communication between cochlear supporting cells, with reduced miR-96 expression accompanying abnormal postnatal cochlear remodeling in Cx26-deficient but not Cx30-deficient models [138]. Complementing this view, transcriptomic analyses of *GJB2* 35delG mutant cochleae have identified region-specific gene-expression changes enriched in pathways related to Ca^2+^ signaling, cell-adhesion molecules, and the synaptic vesicle cycle, arguing that Cx26-related pathology cannot be reduced to a single physiological defect [199].

These regulatory abnormalities are likely to interact with downstream stress pathways that amplify tissue injury over time. Experimental work has shown that partial or complete Cx26 deficiency can be accompanied by oxidative damage, reduced glutathione release, and dysregulation of Nrf2-associated antioxidant programs, supporting the idea that impaired redox resilience is a major component of secondary cochlear vulnerability [101,200]. In parallel, Cx26-null cochleae exhibit a macrophage-dominated local immune response in which CX3CL1, rather than a broad induction of classical inflammatory mediators, appears to play a central role in recruiting immune cells to sites of sensory epithelial degeneration [164]. Other studies further suggest that Cx26 loss is associated with abnormal phasing of apoptosis relative to autophagy, together with defective ATP-dependent Ca^2+^ signaling, indicating that stress-response programs become disordered rather than simply overactivated [201]. Adding another layer of complexity, recent evidence from aging models suggests that endoplasmic reticulum stress may itself accelerate Cx26 degradation through ubiquitination-related mechanisms, raising the possibility of a self-reinforcing loop in which primary connexin deficiency and secondary cellular stress progressively intensify one another [202]. Taken together, these findings support a broader view of *GJB2*-related hearing loss as a dynamic pathogenic process in which non-canonical regulatory disruption and secondary injury amplification help shape phenotypic progression and therapeutic responsiveness.

#### 5.5.2. Anti-Inflammatory and Stress-Modulating Therapeutic Opportunities

Pharmacologic intervention at this stage is best understood not as a substitute for correcting the primary connexin defect, but as an adjunct strategy aimed at dampening the downstream inflammatory and oxidative milieu that amplifies cochlear injury once Cx26 dysfunction has already destabilized the tissue [203,204,205]. In this context, the strongest *GJB2*-specific evidence currently supports glucocorticoid-based immunomodulation [173]. In Cx26-deficient settings, dexamethasone has been shown to reduce macrophage recruitment, limit outer hair cell damage, and improve auditory outcomes, indicating that part of the pathological burden remains pharmacologically modifiable even after the initiating genetic lesion is established. This logic is therapeutically important because it places anti-inflammatory treatment not at the level of developmental rescue or direct blockade of death-execution pathways, but at the level of secondary injury amplification, where excessive immune activation can magnify otherwise localized epithelial damage [206,207].

A parallel rationale applies to redox-directed protection. Cx26 deficiency has been linked to impaired antioxidant resilience, including redox imbalance, reduced glutathione release, and dysregulation of Nrf2-associated defense programs, suggesting that oxidant stress is not merely an incidental accompaniment but a permissive condition for progressive cochlear injury [208]. Although antioxidant therapy has not yet been developed into a mature *GJB2*-specific pharmacologic platform, broader inner-ear studies provide a useful proof of principle that suppressing ROS-associated injury can preserve hair cells and improve hearing outcomes in selected settings [209,210]. N-acetylcysteine is therefore best viewed here not as an established therapy for *GJB2*-related deafness, but as a mechanistically relevant example supporting the broader idea that Cx26-deficient cochleae may benefit from strategies that reinforce redox buffering capacity and interrupt oxidative stress propagation [211,212].

In addition to conventional antioxidant and anti-inflammatory agents, herbal-derived and natural-product-based interventions may provide another exploratory source of stress-modulating strategies. Several compounds or formulations derived from traditional herbal medicine have been investigated in broader sensorineural hearing loss models, where their proposed effects include attenuation of oxidative stress, suppression of inflammatory signaling, modulation of apoptosis, improvement of mitochondrial function, and regulation of cochlear microcirculation [213]. For example, natural compounds such as resveratrol, curcumin, ginsenoside-related compounds, and baicalin have shown otoprotective potential in noise-induced, ototoxic, or age-related hearing loss models [214,215,216,217]. However, there is currently limited direct evidence that these approaches can correct *GJB2*-specific pathogenic mechanisms or restore Cx26-dependent intercellular coupling. Therefore, herbal or natural-product-based therapies should be regarded as adjunctive candidates for modulating secondary cochlear injury rather than as substitutes for genotype-directed rescue. Their potential value in *GJB2*-related hearing loss would require validation in appropriate Cx26-deficient or variant-specific models, with careful attention to dosing, inner-ear delivery, pharmacologic standardization, and safety.

Taken together, current evidence favors a combination framework rather than single-pathway pharmacology. Experimental and clinical studies outside the strict *GJB2* setting have shown that dexamethasone and N-acetylcysteine can provide greater protection together than either agent alone, consistent with the view that inflammatory and oxidative injury are mutually reinforcing rather than independent processes [218,219,220]. This idea is especially relevant to *GJB2*-related hearing loss because recent work further suggests that anti-inflammatory medication may also facilitate restorative approaches: dexamethasone enhanced the effect of AAV-mediated *GJB2* replacement in a conditional Cx26-null model, implying that control of macrophage-associated collateral injury may improve the performance of upstream gene-based interventions [106]. Accordingly, the therapeutic value of this class of treatment lies less in reversing the disease on its own rather than in stabilizing the injured cochlear environment, reducing secondary amplification, and potentially widening the window in which definitive restorative strategies can succeed.

## 6. Carrier Screening and Reproductive Prevention Strategies for *GJB2*-Related Hearing Loss

Given the high carrier frequency of pathogenic *GJB2* variants, a nontrivial proportion of couples will be double carriers and therefore face an elevated probability of having an affected child [221,222]. Accordingly, multiple screening models have been adopted [223]. Preconception or premarital screening aims to identify carrier couples before pregnancy, typically using targeted panels enriched for recurrent DFNB1 alleles or expanded next-generation sequencing panels covering common deafness genes [224]. When both partners carry pathogenic or likely pathogenic DFNB1 variants, counseling can focus on genotype-informed recurrence risk and on reproductive options, including natural conception with newborn testing, prenatal diagnosis, or preimplantation genetic testing where available [225]. Cascade testing in families with an affected child remains another efficient pathway, as it clarifies risk for relatives of reproductive age and supports timely counselling [226].

**Figure 2:**
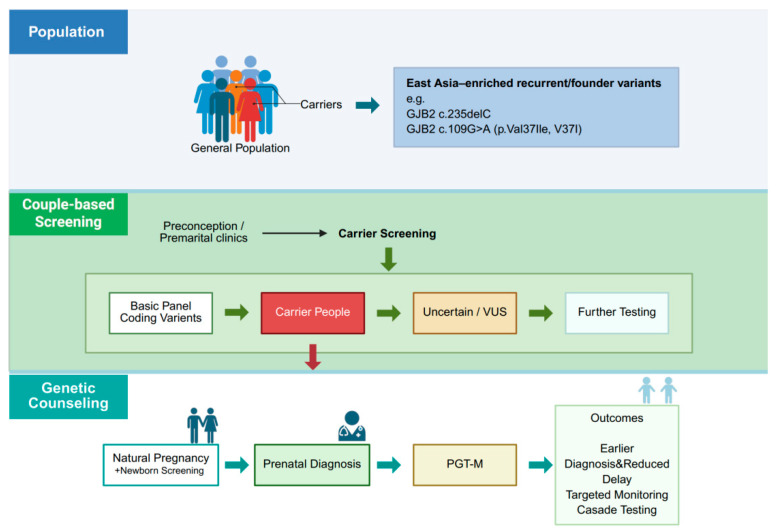
Population-based carrier screening and genetic counseling pathways for *GJB2*-related hearing loss.

Newborn screening provides a complementary entry point for integrating *GJB2* testing into early life care [227]. Adding a small set of recurrent *GJB2* variants to physiological hearing screening can increase the yield of early molecular diagnosis and improve triage [228]. Infants who pass physiological screening but have biallelic genotypes associated with delayed onset or progressive loss can be enrolled into structured audiological follow-up and risk reduction counseling, while those who fail hearing screening and have definitive DFNB1 genotypes can proceed rapidly to confirmatory testing and early habilitation [229]. Newborn results can also inform parental carrier status and future reproductive planning. Screening programs must address genotype–phenotype complexity [230]. Truncating alleles are often associated with severe congenital loss in biallelic states, whereas common hypomorphic missense variants such as p.V37I more often underlie milder, variably penetrant phenotypes, and non-coding or structural lesions can modulate DFNB1 expression [231]. Counseling should therefore communicate a spectrum of likely outcomes and residual uncertainty, rather than implying a binary affected-versus-unaffected prediction (Figure 2) [232].

Beyond clinical and molecular considerations, implementation of *GJB2* carrier screening requires health-system-level evaluation, because the balance among clinical benefit, cost, and feasibility is highly context-dependent [233]. Cost-effectiveness will vary with population carrier frequency, regional variant spectrum, test design, sequencing or genotyping costs, reimbursement policies, counseling capacity, and the availability of downstream reproductive, diagnostic, and early-intervention services. In populations with a limited number of recurrent or founder DFNB1 variants, targeted panels may offer a practical and relatively affordable approach, particularly for premarital or preconception screening [234]. By contrast, broader next-generation sequencing panels can capture allelic heterogeneity and improve diagnostic yield, but they also increase demands for laboratory infrastructure, variant curation, copy number analysis, interpretation of variants of uncertain significance, and post-test counselling [235]. Implementation strategies therefore differ across healthcare systems. Well-resourced settings may integrate carrier testing with expanded reproductive screening or newborn hearing programs, whereas resource-limited settings may prioritize cascade testing, family-history-based testing, or targeted testing in high-prevalence populations [236]. These differences are not merely operational. If testing is accessible mainly to families with higher income, better insurance coverage, urban residence, or access to specialist genetics services, carrier screening may unintentionally widen existing disparities in genetic diagnosis, reproductive counselling, and early hearing intervention [237]. Underrepresentation of certain ancestries in variant databases may further reduce interpretive accuracy and counselling quality. Therefore, population-based *GJB2* screening should be locally adapted, financially accessible, culturally acceptable, and linked to clear referral pathways for genetic counselling, reproductive decision-making, newborn follow-up, and habilitation, rather than implemented as a uniform testing model across all settings.

Finally, assay design and interpretation are critical [238]. Panels limited to common coding variants may miss upstream deletions and other regulatory lesions, leaving apparently monoallelic cases unresolved and underestimating risk. Where founder deletions are prevalent, incorporating dedicated deletion assays and appropriate copy number analysis can materially improve detection. At the same time, cautious interpretation is needed for common noncoding variants with modest effects to avoid unnecessary anxiety [239]. Ethically, programs should emphasize informed consent, reproductive autonomy, equitable access, and the availability of effective rehabilitation, while anticipating that counseling frameworks may evolve as mechanism-based therapies for DFNB1 move closer to clinical application.

## 7. Conclusions and Future Directions

Research on *GJB2*-related hearing loss is shifting from a view centered on a single causative gene defect toward a more integrated understanding of a disease driven by multiple layers of pathogenic processes, and its therapeutic strategies are therefore moving beyond simple etiologic correction toward more refined mechanism-based stratified interventions. Although gene therapy, RNA-based regulation, and protective treatments targeting secondary injury have shown considerable promise, their clinical translation remains constrained by several key challenges, including delivery systems, target-cell specificity, therapeutic timing, and long-term efficacy. Future advances in the treatment of *GJB2*-related hearing loss will depend on integrating molecular diagnosis, mechanistic stratification, and stage-specific intervention strategies, particularly by defining when cochlear architecture, supporting-cell competence, and neural integrity remain sufficiently preserved for gene, RNA, or adjunct pharmacologic therapies to achieve functional benefit.

## Figures and Tables

**Table 1:** Recurrent/founder DFNB1 alleles by population.

Population/Region	Major Recurrent/Founder Alleles	Notes
East Asia [47,48,49,50]	c.235delC c.109G>A (p.Val37Ile) c.299_300delAT c.−23+1G>A (region-specific)	A limited, population-matched panel can capture a substantial proportion of DFNB1 cases; p.Val37Ile is often linked to milder/variable or later-onset hearing loss.
Europe [51]	c.35delG	High-yield first-tier variant in many cohorts; consider reflex testing for DFNB1 structural variants (e.g., *GJB6* deletions) when only one pathogenic *GJB2* allele is detected.
Yakut/Native Siberian groups [52]	c.−23+1G>A (IVS1+1G>A)	Classic founder architecture in Yakut and related groups; emphasizes ancestry-matched panels.
Ashkenazi Jewish/parts of the Middle East [53]	c.167delT	Frequent founder allele; inclusion improves first-tier yield in relevant populations.
South Asia & Romani [54,55]	c.71G>A (p.Trp24Ter; “W24X”)	High carrier frequency reported in some Romani and South Asian subpopulations; useful for targeted screening.
West Africa (e.g., Ghana) [56,57]	c.427C>T (p.Arg143Trp)	A regionally prevalent recurrent allele, highlighting the need for population-tailored screening panels

**Table 4:** Experimental systems used to study *GJB2*-related hearing loss and therapeutic strategies.

Experimental System	Main Applications	Strengths	Limitations	Relevance to Therapeutic Evaluation
Heterologous expression systems, including HEK293/HeLa cells and Xenopus oocytes	Variant-level analysis of Cx26 expression, trafficking, channel permeability, gating, and hemichannel activity	Mechanistically clean; suitable for comparing individual variants; useful for distinguishing loss-of-function, dominant-negative, and gain-of-function effects	Lacks cochlear cellular context, Cx26/Cx30 network organization, developmental timing, and tissue-level physiology	Useful for identifying whether a variant may require expression restoration, trafficking rescue, channel modulation, or mutant-allele suppression
Cochlear-relevant cell systems and iPSC-derived CX26 gap junction-forming cells	Modeling Cx26/Cx30 plaque formation, intercellular coupling, mutation-specific cellular defects, and drug screening	More human-relevant than simple heterologous systems; can support patient-specific or variant-specific testing	Still simplified; does not reproduce mature organ of Corti architecture, lateral wall function, endolymphatic gradients, or auditory output	Useful for early screening of compounds or gene/RNA strategies before tissue-level validation
Ex vivo cochlear organotypic cultures	Early tissue-level assessment of *GJB2* delivery, Cx26 expression restoration, and gap junction coupling	Retains partial immature cochlear architecture and local supporting-cell relationships; useful for proof-of-concept rescue studies	Limited culture duration; lacks mature cochlear mechanics, systemic immune response, endocochlear potential, and long-term auditory readouts	Useful for testing whether early intervention can restore coupling before irreversible degeneration
Murine *GJB2* conditional knockout or knockdown models	Developmental pathology, organ of Corti maturation, sensory epithelial degeneration, and gene supplementation	Preserves cochlear tissue architecture, developmental timing, and auditory physiology; allows ABR, DPOAE, histology, and therapeutic testing	Severe models may overrepresent congenital profound phenotypes; outcomes depend on Cre line, timing, and residual Cx26 expression	Essential for testing therapeutic window, cell specificity, dosing, toxicity, and functional rescue
Murine knock-in, dominant-negative, or inducible models	Patient-relevant variants, progressive phenotypes, mature-stage Cx26 loss, and dominant mechanisms	Better suited for genotype-specific mechanisms and later intervention windows; useful for dominant-negative or gain-of-function variants	May not capture full human allelic diversity; model construction and phenotype severity vary	Important for testing allele-specific editing, RNA interference, hemichannel blockade, and late-stage protective strategies
Inner-ear organoids	Humanized disease modeling, scalable drug screening, and future patient-specific therapeutic testing	Derived from pluripotent stem cells; potentially useful for human genetic background and personalized screening	Immature; incomplete cochlear supporting-cell network, lateral wall, stria vascularis, endolymphatic physiology, tonotopy, immune context, and functional auditory output	Promising complementary platform, but not yet sufficient to replace murine or in vivo validation

**Table 5:** Representative gene supplementation studies for upstream rescue in *GJB2*-related hearing loss.

Year	Study	Model	Treatment Window	Delivery Route	Principal Target Cells	Key Findings
2011 [97]	Crispino et al.	Cx26 conditional deletion, organ of Corti explant culture	Early postnatal tissue, ex vivo	Ex vivo viral transduction in cochlear explants	Non-sensory epithelium and supporting-cell network	Restored Cx26 expression and improved intercellular coupling, providing proof of concept that *GJB2* supplementation can re-engage epithelial gap junction function.
2014 [99]	Yu et al.	Conditional *GJB2* loss in supporting cell lineages	Neonatal, around birth	Cochlear local injection targeting scala media	Supporting cells and adjacent epithelial cells	Re-established the gap junction network and reduced epithelial injury, but hearing recovery remained limited or inconsistent.
2015 [132]	Iizuka et al.	Cx26 conditional loss model	Perinatal versus adult comparison	Round-window-based local delivery	Supporting cell enriched patterns in the cochlea	Perinatal delivery improved ABR thresholds and preserved cochlear architecture, whereas adult-stage treatment showed little benefit, supporting a narrow developmental rescue window.
2021 [133]	Guo et al.	Inducible *GJB2* deficiency model	More mature stage, postnatal weeks	Round-window-related local cochlear delivery	Supporting cells with unintended inner hair-cell transduction	Restored Cx26 signal in supporting cells but failed to improve hearing; ectopic inner hair cells expression was associated with hair cell loss, highlighting the need for strict cell specificity.
2025 [106]	Wang et al.	Conditional Cx26 deficiency model	Neonatal	Local cochlear delivery	Supporting cell-targeted expression	Vector delivery triggered marked immune activation and could compromise hearing in wild-type ears; adjunct anti-inflammatory treatment improved tolerability and functional outcome.
2025 [103]	Ivanchenko et al.	DFNB1 mouse models with non-human primate validation	Early postnatal	Round window local delivery	Expression constrained to physiologic cochlear cell populations	Regulatory-element-constrained expression improved cochlear pathology and hearing in DFNB1 models, while showing appropriate localization and minimal threshold disturbance in non-human primate cochlea.

## Data Availability

No new data were created or analyzed in this study. Data sharing is not applicable. All figures in the manuscript were designed by the authors and created using BioRender.com under an appropriate publication license. No previously published third-party figures were reproduced or adapted.
